# Genome-wide association mapping of septoria nodorum blotch resistance in Nordic winter and spring wheat collections

**DOI:** 10.1007/s00122-022-04210-z

**Published:** 2022-09-23

**Authors:** Min Lin, Andrea Ficke, Jon Arne Dieseth, Morten Lillemo

**Affiliations:** 1 Department of Plant Sciences, Norwegian University of Life Sciences, Post Box 5003, NO-1432 ÅS, Norway; 2 Division of Biotechnology and Plant Health, Norwegian Inst. of Bioeconomy Research, P.O. Box 115, NO-1431 ÅS, Norway; 3 Graminor, AS, Bjørke Gård, Hommelstadvegen 60, NO-2322 Ridabu, Norway

## Abstract

**Key message:**

A new QTL for SNB, *QSnb.nmbu-2AS*, was found in both winter and spring wheat panels that can greatly advance SNB resistance breeding

**Abstract:**

Septoria nodorum blotch (SNB), caused by the necrotrophic fungal pathogen *Parastagonospora nodorum*, is the dominant leaf blotch pathogen of wheat in Norway. Resistance/susceptibility to SNB is a quantitatively inherited trait, which can be partly explained by the interactions between wheat sensitivity loci (*Snn*) and corresponding *P. nodorum* necrotrophic effectors (NEs). Two Nordic wheat association mapping panels were assessed for SNB resistance in the field over three to four years: a spring wheat and a winter wheat panel (*n* = 296 and 102, respectively). Genome-wide association studies found consistent SNB resistance associated with quantitative trait loci (QTL) on eleven wheat chromosomes, and ten of those QTL were common in the spring and winter wheat panels. One robust QTL on the short arm of chromosome 2A, *QSnb.nmbu-2AS*, was significantly detected in both the winter and spring wheat panels. For winter wheat, using the four years of SNB field severity data in combination with five years of historical data, the effect of *QSnb.nmbu-2AS* was confirmed in seven of the nine years, while for spring wheat, the effect was confirmed for all tested years including the historical data from 2014 to 2015. However, lines containing the resistant haplotype are rare in both Nordic spring (4.0%) and winter wheat cultivars (15.7%), indicating the potential of integrating this QTL in SNB resistance breeding programs. In addition, clear and significant additive effects were observed by stacking resistant alleles of the detected QTL, suggesting that marker-assisted selection can greatly facilitate SNB resistance breeding.

**Supplementary Information:**

The online version contains supplementary material available at 10.1007/s00122-022-04210-z.

## Introduction

Wheat (*Triticum aestivum* L.) is the most commonly cultivated crop worldwide (FAO 2020). Because of the enormous efforts on wheat breeding and optimization of field management, global wheat yield increases continuously and reached 3.5 tonnes per hectare on average in 2020 (FAO 2020). However, grain yield can be greatly reduced by various wheat pests and pathogens. Septoria nodorum blotch (SNB) is one of the most devastating fungal diseases of wheat which reduces both grain yield and quality and can cause up to 30% yield loss under warm and humid conditions (Bhathal et al. 2003). The causal agent of SNB is the necrotrophic pathogen *Parastagonospora nodorum*. By secreting necrotrophic effectors (NEs), *P. nodorum* can trigger plant cell death and take up nutrients from dying host tissues to accelerate infection (Friesen and Faris 2012). In contrast to the ‘gene-for-gene model’ for the interactions between biotrophic pathogens and their hosts (Flor 1956), *P. nodorum* interacts with wheat in an inverse gene-for-gene manner (Friesen and Faris 2012; Friesen et al. 2007). Wheat sensitivity loci (*Snn*) interact with corresponding NEs produced by *P. nodorum*, which leads to increased pathogenicity of SNB. Six *P. nodorum* NEs have been characterized to interact with nine wheat susceptibility loci, reviewed by Peters Haugrud et al. (2022). So far, five *P. nodorum* NEs (ToxA, Tox1, Tox3, Tox267, and Tox5) and three host susceptibility loci (*Tsn1*, *Snn1*, and *Snn3-D1*) have been characterized at the gene sequence level (Faris et al. 2010; Liu et al. 2009, 2012; Shi et al. 2016; Richards et al. 2022; Kariyawasam et al. 2022; Zhang et al. 2021). Resistance to SNB is known as a polygenetic trait involving many genes with minor effects (Fried and Meister 1987; Wicki et al. 1999). The increasing understanding of the NE-*Snn* interactions indicates that wheat susceptibility to SNB is also a quantitative trait, and the effects of NE-*Snn* interactions vary from additive to epistatic (Peters Haugrud et al. 2019). Fungicides are widely and regularly applied for SNB management (Ficke et al. 2018; Ruud and Lillemo 2018). However, the potential risk of fungicide resistance and the environmental concerns of fungicide application are increasing. Improving cultivar resistance to SNB is essential as it helps to manage this plant disease in a more effective and sustainable manner.

Choosing the appropriate QTL for marker-assisted selection (MAS) is a challenge when we want to improve SNB resistance. Resistance QTL characterized by seedling experiments are not necessarily relevant for adult-plant resistance, and only a few *Snn* loci have shown effects in field studies (Francki 2013; Friesen et al. 2009; Phan et al. 2016; Ruud et al. 2019, 2017; Ruud and Lillemo 2018; Lin et al., 2020; Lin & Lillemo 2021). In addition, *P. nodorum* reproduces both asexually and sexually, and the rapid co-evolution of the pathogen population makes breeding of cultivars with durable resistance very difficult (McDonald and Linde 2002). Genome-wide association studies (GWAS) have been widely used for identifying marker-trait associations (MTAs) of polygenetic traits in plants (Gupta et al. 2014; Gurung et al. 2014; Kidane et al. 2017; Korte and Farlow 2013; Xiao et al. 2017). However, most GWAS on SNB resistance were based on seedling resistance (Adhikari et al. 2011; Gurung et al. 2014; Liu et al. 2015; Phan et al. 2018). The first GWAS for SNB adult-plant leaf blotch resistance was done by Ruud et al. (2019). Robust QTL for adult-plant resistance was identified on eight chromosomes, using a subset of 121 lines of the Nordic spring wheat association mapping panel (Ruud et al. 2019). A QTL on the long arm of chromosome 2D turned out to be the most robust QTL for adult-plant resistance. It was detected in six out of seven years of testing, and the haplotype analysis confirmed the importance of this QTL (Ruud et al. 2019).

The objectives of this study were: (1) to discover robust QTL for adult-plant SNB resistance by association analysis using a winter wheat association panel; (2) to compare QTL discovered in a previous GWAS field study by Ruud et al. (2019) using an enlarged spring wheat panel; and (3) to compare SNB resistance- associated QTL between spring and winter wheat.

## Materials and methods

### Plant material and genotyping

The NMBU spring wheat panel of 296 spring wheat lines and the NMBU winter wheat panel of 102 winter wheat lines include current and historical Nordic wheat cultivars, breeding lines and selected crossing parents and resistance sources of international origin. The spring and winter wheat panels were scored for adult- plant leaf blotch resistance in the field. A subset of 121 spring wheat lines was genotyped by Illumina 90 K wheat SNP chip (Wang et al. 2014) and tested for SNB leaf blotch from 2010 to 2016, as described by Ruud et al. (2019). This study used an expanded panel of 296 spring wheat and 102 winter wheat lines, which were genotyped by the TraitGenetics 25 K. In addition, a few KASP markers designed for significant SNB QTL detected in the previous GWAS by Ruud et al. (2019) and SNB resistance-related microsatellite (SSR) markers used in the same study were also included. SSR markers were converted to be biallelic. KASP and SSR markers were first placed on an artificial chromosome. Significant markers from the artificial chromosome were then placed on the map putatively according to physical map positions and LD results. Monomorphic markers and minor alleles with lower than 5% allele frequency were filtered out. The final GWAS analysis contained 18,570 markers (SNP and SSR) for the winter wheat panel, and 18,578 markers (SNP and SSR) for the spring wheat panel.

### Field testing

For the spring wheat panel, field testing was conducted for three years from 2016 at Vollebekk research station in Ås, Norway, using alpha lattice designs with three replicates. The winter wheat panel was tested in the field at the same location for four years since 2016, using alpha lattice design with three replicates. Field evaluation and field control methods were previously described by Ruud et al. (2019). Briefly, naturally *P. nodorum-*infected straw was used as inoculum, and mist irrigation was carried out to enhance infection (as described by Ruud et al., 2019). Leaf blotch scorings were carried out two to three times, assessing the percentage of diseased leaf area in each hillplot canopy, starting when the most susceptible lines reached 60–70% disease severity (around GS 83). The subsequent scorings were carried out with approximately one-week time intervals. As correlated traits to SNB severity, plant height (cm) and days to heading (days) were also measured as described by Lin et al. (2020).

### Statistical analysis

‘Plant height’ and ‘days to heading’ were used as covariates in regression to obtain the corrected disease severity of leaf blotch for the spring wheat panel as described by Ruud et al. (2019). No significant association was found between leaf blotch and ‘plant height’ in most years for the winter wheat panel. Therefore, only ‘days to heading’ was used as covariate for correcting winter wheat disease severity. The across-year means of corrected disease severity for each genotype were calculated using environments (year) as random effect and genotype as fixed effect by PROC MIXED implemented in SAS 9.4 (SAS Institute, Inc.). Pearson correlation coefficients were calculated by R package `Hmisc` (Harrell 2019). Variance components were calculated by fitting genotype and environment as random effects using R package lme4 (Bates et al. 2015). Broad-sense heritability was calculated using formula ${h}^{2}={\sigma }_{\mathrm{g}}^{2}/({\sigma }_{\mathrm{g}}^{2}+{\sigma }_{\mathrm{E}}^{2}/y+{\sigma }_{\mathrm{e}}^{2}/ry)$, where ${\sigma }_{\mathrm{g}}^{2}$ is the genetic variance, ${\sigma }_{\mathrm{E}}^{2}$ is the environmental variance, ${\sigma }_{\mathrm{e}}^{2}$ is the error variance, *y* is the number of environments, and r is the number of replicates.

### Linkage disequilibrium and population structure

The pairwise linkage disequilibrium (LD) was calculated based on the squared frequency correlation (*r*^2^) (Hill and Weir 1988) using functions implemented in TASSEL v.5.2.48 (Bradbury et al. 2007). LD analysis for markers on single chromosomes used the full-matrix option in TASSEL (Bradbury et al. 2007). The population structures in the spring and winter wheat panels were investigated previously by Branchereau (2018) using Bayesian clustering in the software STRUCTURE v2.3.4 (Pritchard et al. 2000). It showed that both panels could be divided into two subpopulations, which largely followed the genetic origin of the lines. For the spring wheat panel, the division was between lines of Nordic origin (subpopulation 1) and exotic lines mainly from CIMMYT and China (subpopulation 2). For the winter wheat panel, the first subpopulation consisted of mainly German and UK wheat lines, while the second was composed of lines from Norway and Sweden.

### Association analysis

Association analyses were done separately for the spring and winter wheat panels. Two models implemented in the R package GAPIT3 (Wang & Zhang et al. 2021) were used for association analyses of SNB. The first was the mixed linear model (MLM) + kinship matrix (K) + Principal component (PC) (Yu et al. 2006), which was used in previous GWAS by Ruud et al. (2019). The second was the FarmCPU (fixed and random model circulating probability unification) model (Liu et al. 2016). In comparison to MLM, instead of using population structure or principal components and kinship as covariates, FarmCPU used all associated markers in a fixed-effect model and also optimized the associated markers in a separate random-effect model, which controlled both false positives and false negatives, and also enabled fast computation. QQ plots from both models were inspected and compared. The FarmCPU model showed better fits to our phenotypic data and QTL showed higher significance (Fig. S1 and S2). Therefore, only results analyzed by the FarmCPU model were selected for further analysis. Since the Bonferroni correction would be a too strict criterion for declaring significant QTL for a highly quantitative trait like SNB field resistance, we applied the 0.1 percentile of the *p*-value distribution as an exploratory significance threshold to detect putative QTL (Chan et al. 2010), as used by the previous study by Ruud et al (2019). QTL were considered as robust when associated markers met the more stringent − log_10_(*p*) threshold of 4.0 in at least one environment, as well as being detected by the across-year mean by either threshold used in this study. In addition, the Quantile–Quantile (QQ) plots were inspected to identify the level at which the observed p-values started to deviate from the expected values under the null hypothesis. The databases https://triticeaetoolbox.org and http://www.cerealsdb.uk.net were used for obtaining SNP marker sequences. Physical map positions of markers on the wheat reference genome IWGSC RefSeq v1.0 (International Wheat Genome Sequencing et al. 2018) were obtained from the database https://urgi.versailles.inra.fr/blast/?dbgroup=wheat_iwgsc_refseq_v1_chromosomes&program=blastn. Significant markers were considered belonging to the same QTL if markers were located within the 10-Mbp interval range or showed high LD (*R*^2^ > 0.8) with each other.

### Haplotype analysis

One stable field-resistance QTL on chromosome 2A identified from both the winter wheat and the spring wheat panel was selected for haplotype analysis. Two markers *Excalibur_c41459_96* and *BS00022760_51,* were selected for the haplotype analysis according to the criteria that markers were above the -log10(p) 4.0 threshold in at least one environment while genotypic data were available for both spring wheat and winter wheat panels (Tables S1 and S2). Pairwise comparison of corrected disease severities between haplotypes was conducted using the Wilcoxon test implemented in *R* package `ggpubr` (Kassambara 2020). Around 50 lines of the winter wheat panel from 2010 to 2015 (except 2013), and around 150 lines of the spring wheat panel from 2014 to 2015 were tested for leaf blotch resistance in the field (data from Ruud et al, 2019). Those historical phenotypic data were used in the haplotype analysis to confirm the haplotype effects caused by this 2AS QTL.

### Stacking resistant alleles

Six robust SNB QTL were selected for the spring and winter wheat panels, to investigate the effect of stacking resistance alleles (Table S3). The resistant allele was determined by comparing the mean of corrected disease severity between alleles using the Wilcoxon test implemented in *R* package `ggpubr` (Kassambara 2020) (Fig. S3 and S4). Wheat lines were grouped by the number of resistant alleles they contained, and Tukey`s HSD test (*p* < 0.05) implemented in the R/multcomView package (Graves 2015) was used to compare whether there were significant differences of mean disease severities between groups. As differences between alleles were not significant for marker *BS00078784_51* in the spring wheat panel (Fig. S3), this marker was excluded from the allele stacking analysis, and the total number of groups for the spring wheat panel was six instead of seven, in comparison to winter wheat.

## Results

### Phenotypic evaluation

Variations in SNB resistance were observed in both panels in all tested years (Fig. S5). For the spring wheat panel, SNB disease severities of different genotypes varied from 3 to 94% in 2016, 7 to 91% in 2017 and 12 to 90% in 2018, while for the winter wheat, the disease severity varied from 14 to 78% in 2016, 14 to 65% in 2017, 16 to 69% in 2018, and 8 to 60% in 2019. As shown by Table 1, both ‘plant height’ and ‘days to heading’ were significantly correlated with leaf blotch severity for the spring wheat panel. For the winter wheat panel, ‘days to heading’ was significantly correlated with leaf blotch severity while ‘plant height’ was only significantly correlated with leaf blotch in 2016 (Table 1). However, the significance level was relatively low (*p* = 0.02), and it was probably due to lodging which was positively correlated with the disease severity that year (data not shown). Corrected disease severities were also highly significantly correlated between environments for both the spring and winter wheat panels (*p* < 0.0001) (Table 2). The heritability of leaf blotch resistance across years was 0.84 for the spring and 0.80 for the winter wheat panel.

**Table 1:** Pearson`s correlation coefficient between leaf blotch severity and plant height (PH) and days to heading (DH) for each year of the winter and spring wheat panels

Year	PH	DH
2016 winter	0.21*	− 0.47***
2017 winter	− 0.03	− 0.71***
2018 winter	− 0.04	− 0.46***
2019 winter	0.02	− 0.36***
2016 spring	− 0.26***	− 0.55***
2017 spring	− 0.12*	− 0.63***
2018 spring	− 0.51***	− 0.67***

***P < 0.0001, *P < 0.05

**Table 2:** Pearson`s correlation coefficients of corrected leaf blotch severities between years for winter and spring wheat panels

	Winter 2016	Winter 2017	Winter 2018	Spring 2016	Spring 2017
Winter 2017	0.57***	–	–	–	–
Winter 2018	0.44***	0.42***		–	–
Winter 2019	0.73***	0.57***	0.49***	–	–
Spring 2017	–	–	–	0.60***	–
Spring 2018	–	–	–	0.61***	0.58***

***P < 0.0001

### Association mapping of the spring wheat panel

**Fig. 1:**
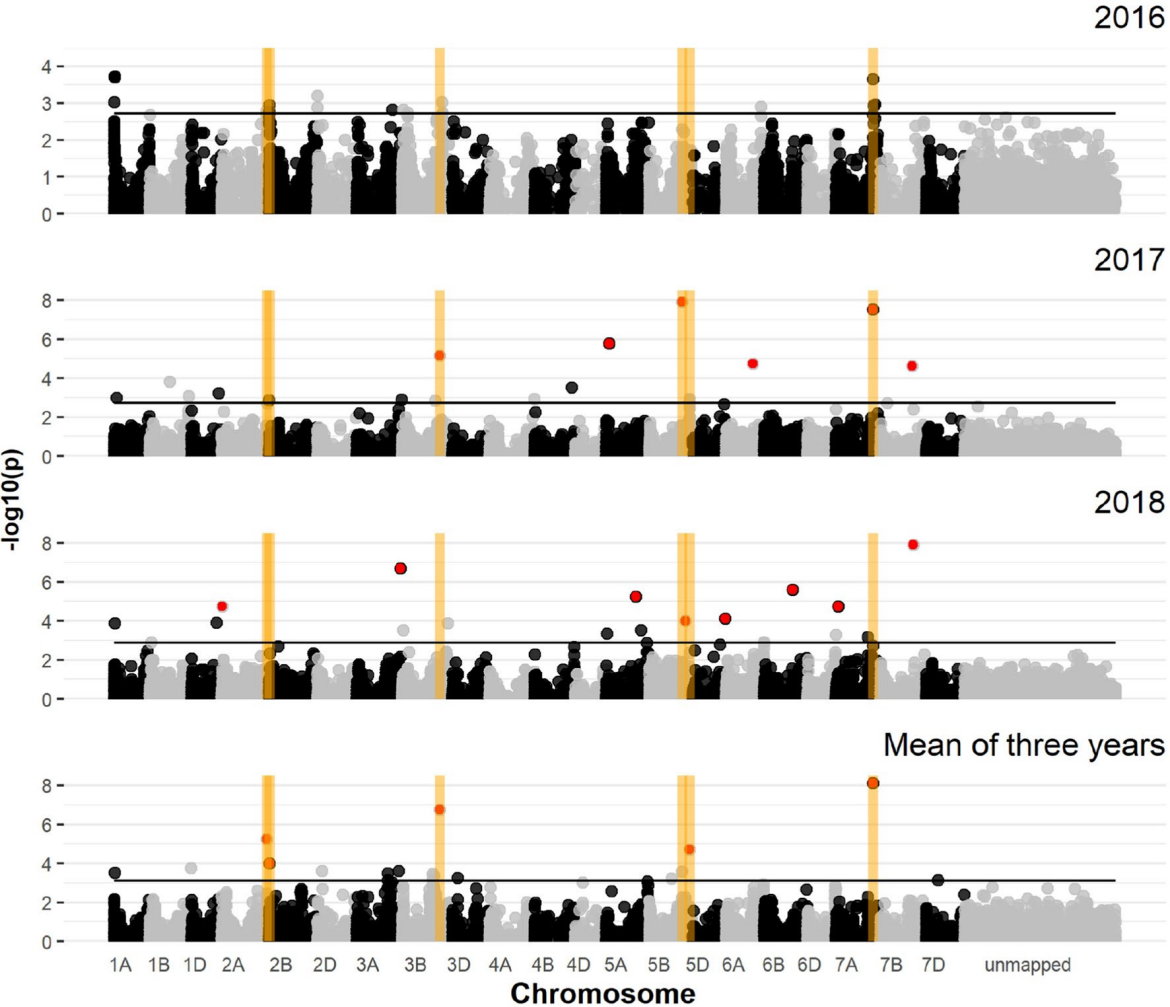
Manhattan plots of marker-trait associations for corrected SNB disease severity in the spring wheat panel. From top to bottom are Manhattan plots for the years 2016, 2017, 2018, and mean of the three years. The 0.1 percentile threshold is indicated as a horizontal line in each subplot. Dots above the threshold indicate significant markers; red dots indicate markers above the − log_10_(*p*) 4.0 threshold. Important QTL (significant in at least two environments or one year and mean across years) are labeled with yellow rectangles

The exploratory − log_10_(*p*) threshold for the spring wheat panel varied between 2.72 and 3.11 (Table S4). In total, 50 marker-trait associations (MTAs) were detected on all chromosomes except chromosome 4D (Table S1, Fig. 1). Out of these, six QTL on chromosomes 2A, 2B, 3B, 5B, and 7A met the more stringent − log_10_(*p*) threshold of 4.0 in at least one environment in addition to being detected in the mean data across years. These were considered as robust QTL. The QTL *QSnb.nmbu-2AL* on chromosome 2AL was identified in year 2016 as a putative QTL while being detected as a robust QTL by the across-year mean. The peak marker *RAC875_c20979_234* (− log10(*p*) = 5.25) was located at 742 Mbp of the chromosome 2A physical map (Table S1). Another QTL *QSnb.nmbu-2BS* on the short arm of chromosome 2B (1–4 Mbp) was detected as a putative QTL in 2016 and above the 4.0 threshold by the across-year mean. Another robust QTL, *QSnb.nmbu-3BL.1,* was identified on the long arm of chromosome 3B (618 Mbp), and single marker *wsnp_JD_c5643_6802088* was detected above the stringent threshold in both year 2017 and the across-year mean. The *Tsn1*-associated QTL *QSnb.nmbu-5BL.3* on chromosome 5B was significantly detected in 2017 (− log10(*p*) = 7.92) and the across year mean (− log10(*p*) = 3.60) (Fig. 1), Another robust QTL *QSnb.nmbu-5BL.4* on chromosome 5B was located at 662–668 Mbp, which was detected as a putative QTL in 2017 and above the 4.0 threshold by the across-year mean. The last robust QTL, *QSnb.nmbu-7AL,* was located on the long arm of chromosome 7A (611–618 Mbp), which was significantly detected in the years 2016 (− log10(*p*) = 3.66), 2017 (− log10(*p*) = 7.51), and the across year mean (− log10(*p*) = 8.11).

In addition to these robust QTL, two consistent QTL were detected as putative QTL in at least two environments. *QSnb.nmbu-1AS.2* on chromosome 1A (14 Mbp) was detected with the 0.1 percentile criterion in the years 2016, 2018, and the across-year mean. The other consistent QTL, *QSnb.nmbu-3BS,* was detected at 18–22 Mbp on chromosome 3B with the 0.1 percentile criterion in both years 2016 and 2018.

### Association mapping of the winter wheat panel

**Fig. 2:**
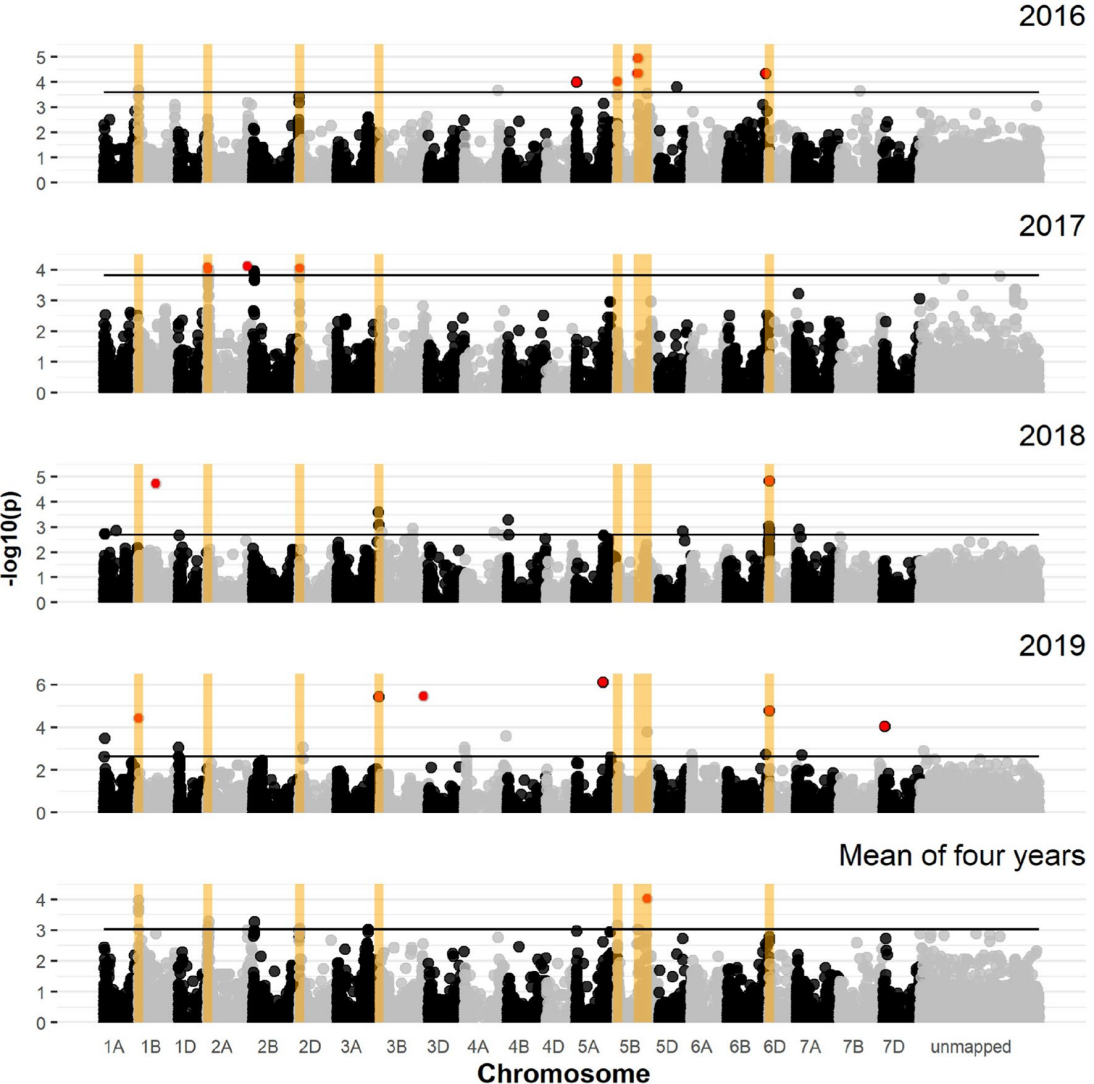
Manhattan plots of marker-trait associations for corrected SNB disease severity in the winter wheat panel. From top to bottom are Manhattan plots for the years 2016, 2017, 2018, 2019 and the mean of four years. The 0.1 percentile threshold is indicated as a horizontal line in each subplot. Dots above the threshold indicate significant markers; red dots indicate markers above the − log_10_(*p*) 4.0 threshold. Important QTL (significant in at least two environments or one year and mean across years) are labeled with yellow rectangles

For the winter wheat panel, the exploratory − log_10_(*p*) threshold for each environment (year) ranged from 2.63 to 3.82 (Table S4), and yielded a total of 41 SNB-associated MTAs, on all chromosomes expect 3D, 4D, and 6D (Fig. 2, Table S2). Out of these, six QTL met the more stringent − log_10_(*p*) threshold of 4.0 in at least one environment in addition to being detected in the mean across years. One consistent QTL was located on chromosome 1B, one on 2A, one on 2D, and three on 5B (Table 3, Fig. 2). The QTL *QSnb.nmbu-1BS* was located at 0.7–4 Mbp on the physical map, which was significantly detected above the − log10(*p*) 4.0 threshold in 2019, and was detected as a putative QTL in 2016 and the across-year mean. The QTL *QSnb.nmbu-2AS* on chromosome 2A was located at 3.5–24 Mbp on the physical map. The QTL interval exceeded the 10 Mbp distance but the significant markers were in high LD. This QTL was detected above the stringent threshold in 2017 while being detected as a putative QTL by the across-year mean (Table 3). Another robust QTL, *QSnb.nmbu-2DS,* located on the short arm of chromosome 2D (8 Mbp), was significantly detected above the 4.0 threshold in 2017 and above the exploratory threshold by the mean across years, likely caused by the *Snn2* locus (Friesen et al. 2007). The *Snn3* KASP marker *BS00091519_51* (Ruud et al. 2017), located at 6.6 Mbp on chromosome 5B was above the − log_10_(*p*) threshold of 4.0 in 2016 as well as being detected as a putative QTL *QSnb.nmbu-5BS.1* by the across-year mean. The second QTL on chromosome 5B*, QSnb.nmbu-5BL.1,* was located at 350–369 Mbp. The physical map interval also exceeded 10 Mbp, but the significant markers were in high LD. This QTL was above the stringent threshold of 4.0 in 2016, while being detected as a putative QTL in 2019 and the across-year mean. The third QTL on chromosome 5B was located at 514 Mbp and the same marker *IAAV5683* was detected above the − log_10_(*p*) threshold of 4.0 by the across-year mean and as a putative QTL in 2019.

**Table 3:** Consistent *P. nodorum* resistance/sensitivity related QTL detected by GWAS analysis from field trials

QTL	Trials	Total number of MTAs	Flanking markers	Position (Mbp)	− log10 (*p*)	References
*QSnb.nmbu-1AS.1*	2016^S^,2019^W^	2	RAC875_c42700_264 and GENE-0014_822	1	2.59–3.02	(Abeysekara et al. 2009)
*QSnb.nmbu-1AS.2*	2016^S^,2018^WS^,2019^W^, across year mean^S^	7	AX-110004070 and RAC875_c23587_271	9–14	2.74–3.88	(Ruud et al. 2019)
*QSnb.nmbu-1BS*	2016^W^, 2019^W^, across year mean^W^	3	fcp618 and AX-95154820	1–4	3.61–4.39	(Liu et al. 2004; Reddy et al. 2008)
*QSnb.nmbu-2AS*	2017^W^, 2018^S^, across year mean^W^	15	Excalibur_c15379_1305 and TA003766-0683	4–24	3.06–4.75	(Francki et al. 2018; Lin et al. 2020; Phan et al. 2016)
*QSnb.nmbu-2AL*	2016^S^, across year mean^S^	2	RAC875_c20979_234 and AX-94646265	742–743	2.79–5.25	(Hu et al. 2019)
*QSnb.nmbu-2BS*	2016^S^, 2017^S^, across year mean^S^	5	wsnp_Ex_c326_636368 and AX-158575300	1–4	2.75–4.00	This study
*QSnb.nmbu-2DS*	2016^S^, 2017^W^, across year mean^W^	4	AX-95146929 and Excalibur_c25599_358	2–8	3.06–4.04	(Friesen et al. 2009)
*QSnb.nmbu-3AL*	2018^WS^, 2019^W^	4	AX-110989461 and AX-158523630	722–724	3.05–6.71	This study
*QSnb.nmbu-3BS*	2016^S^, 2018^WS^	3	AX-158523648 and AX-158579373	17–22	2.81–3.53	(Jighly et al. 2016)
*QSnb.nmbu-3BL.1*	2017^S^, across year mean^S^	1	wsnp_JD_c5643_6802088	618	5.15–6.75	This study
*QSnb.nmbu-3BL.2*	2018^S^, 2019^ W^	2	wsnp_Ex_c8208_13870372 and BS00077967_51	742–750	3.88–5.58	This study
*QSnb.nmbu-5AS*	2016^ W^, 2018^S^	2	GENE-3324_338 and AX-95106872	4–10	3.35–4.01	This study
*QSnb.nmbu-5BS.1*	2016^ W^, across year mean^W^	1	BS00091519_51	7	3.15–4.02	(Friesen et al. 2007; Ruud et al. 2019)
*QSnb.nmbu-5BL.1*	2016^W^, 2019^W^, across year mean^WS^	13	Kukri_c23070_350 and Tdurum_contig12540_72	350–370	3.04–4.94	This study
*QSnb.nmbu-5BL.2*	2019^W^, across year mean^W^	1	IAAV5683	514	3.72–4.03	(Lin et al. 2021)
*QSnb.nmbu-5BL.3*	2017^S^, across year mean^S^	2	fcp001 and AX-89422431	546–547	3.60–7.92	(Friesen et al. 2006)
*QSnb.nmbu-5BL.4*	2017^S^, across year mean^S^	4	AX-94559013 and BS00078784_51	662–668	2.81–4.72	This study
*QSnb.nmbu-6BL*	2018^W^, 2019^W^	4	Tdurum_contig28247_226 and Excalibur_c55484_393	718–721	3.04–4.82	This study
*QSnb.nmbu-7AS*	2018^WS^	2	AX-158553506 and AX-158590739	33–37	2.92–4.73	This study
*QSnb.nmbu-7AL*	2016^S^, 2017^S^, across year mean^S^	3	Tdurum_contig62357_527 and AX-94387533	611–618	2.93–8.11	This study

^*S*^ Spring wheat panel, ^*W*^ winter wheat panel, *MTAs* marker-trait associations, Positions: physical map positions of QTL in Mbps on IWGSC RefSeq v1.0; References: studies where QTL were found containing overlapping intervals with QTL identified by our GWAS on IWGSC RefSeq v1.0

Three additional QTL were also considered important as they were detected above the 0.1 percentile exploratory threshold in two environments (years). These QTL were located on chromosomes 1A, 3A, and 6B (Table 3, Fig. 2). The QTL *QSnb.nmbu-1AS.2* detected on chromosome 1A was located at 9–13 Mbp on the physical map, which was above the exploratory 0.1 percentile threshold in both 2018 and 2019. The QTL *QSnb.nmbu-3AL,* identified on chromosome 3AL, located at 722 Mbp, which was above the stringent threshold in 2019 while above the 0.1 percentile threshold in 2018. The QTL *QSnb.nmbu-6BL* on chromosome 6B was located at 718–721 Mbp, which was above the stringent threshold of 4.0 in both 2018 and 2019.

### Common QTL between spring and winter wheat

By sorting the physical map positions of the significant markers, ten common QTL were found between the spring and winter wheat panels (Table 3). Two common QTL were detected on the short arm of chromosome 1A. The first one, *QSnb.nmbu-1AS.1,* was located at 1 Mbp, which was identified as a putative QTL by spring wheat in 2016 and by winter wheat in 2019 (Table 3). The second QTL *QSnb.nmbu-1AS.2* on chromosome 1A was located at 9–14 Mbp (Table 3), which was above the exploratory threshold in winter wheat in the years 2018 and 2019, and in spring wheat in the years 2016, 2018, and the across-year mean. Interestingly, although not being consistently detected by spring wheat, the robust QTL *QSnb.nmbu-2AS,* identified on chromosome 2A by the winter wheat panel was also significantly detected (− log10(*p*) = 4.75) by the spring wheat panel in 2018. In addition, the *Snn2* locus-related QTL *QSnb.nmbu-2DS* identified in winter wheat was also detected as a putative spring wheat QTL in 2016. Moreover, the consistent winter wheat QTL *QSnb.nmbu-3AL* on chromosome 3A, was also significant in the spring wheat panel in 2018 (− log10(*p*) = 6.70). Two common QTL were identified on chromosome 3B. The first one was *QSnb.nmbu-3BS* located at 17–22 Mbp, which was consistently detected as a putative spring wheat QTL in 2016 and 2018. A significant marker located at 17 Mbp on chromosome 3B was also identified in the winter wheat panel in year 2018. The second common QTL, *QSnb.nmbu-3BL.2* on chromosome 3B, was located on the long arm (742–750 Mbp), which was significant in year 2019 (− log10(*p*) = 5.58) in the winter wheat panel, while being detected as a putative spring wheat QTL in 2018 (− log10(*p*) = 3.88). *QSnb.nmbu-5AS*, on chromosome 5A, was located at 4–10 Mbp, which was above the 4.0 threshold in the winter wheat panel in 2016 and above the 0.1 percentile threshold in the spring wheat panel in 2018. As mentioned in the previous section, the robust QTL *QSnb.nmbu-5BL.1* was first identified in winter wheat on chromosome 5B (350–369 Mbp). A putative QTL on chromosome 5B (370 Mbp, − log10(*p*) = 3.23), detected by the across-year mean using the spring wheat panel, was located quite close to this QTL. The last common QTL, *QSnb.nmbu-7AS* located on chromosome 7A (33–37 Mbp), was identified above the stringent threshold in spring wheat in 2018 while being detected as a putative QTL in winter wheat during the same year.

### Haplotype analysis

**Fig. 3:**
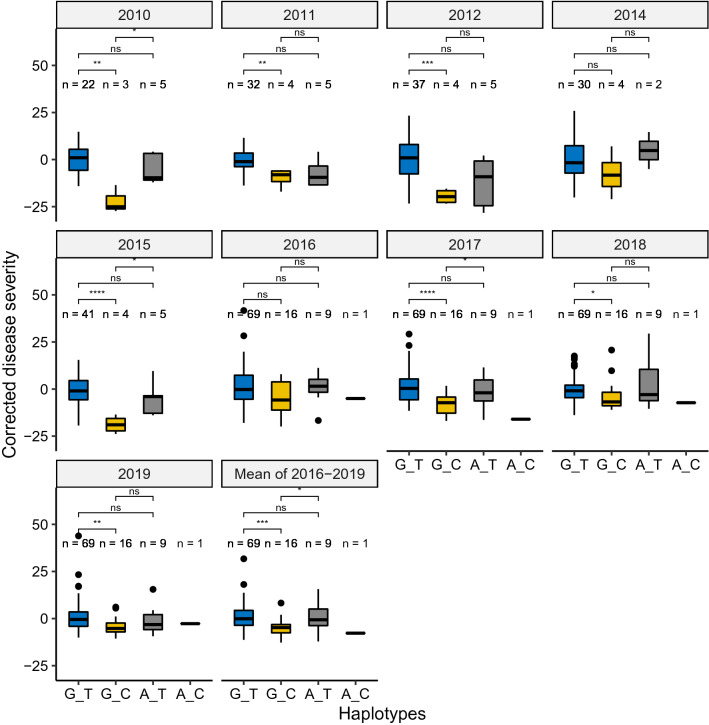
Haplotype analysis of the QTL *QSnb.nmbu-2AS* in the winter wheat panel over nine years field trials and the mean of four years from 2016 to 2019 (Bottom right) determined by the Wilcox test. ns: *p* > 0.05; *: *p* <  = 0.05; **: *p* <  = 0.01; ***: *p* <  = 0.001

Four haplotypes were constructed based on the combination of alleles from two markers of the *QSnb.nmbu-2AS* (Fig. 3). For winter wheat, in total, 9 years of leaf blotch data of the winter wheat panel were used for haplotype analysis of the QTL *QSnb.nmbu-2AS*. Significant differences of corrected leaf blotch severities were detected between resistant and susceptible haplotypes in seven out of nine years of testing and also the across-year mean from 2016 to 2019 (Fig. 3, Table S5). The susceptible haplotype “G_T” always had higher disease severity compared to the resistant haplotype “G_C”. In addition, in 2010, 2015, 2017, and the across-year mean from 2016 to 2019, significant differences were also detected between resistant haplotype “G_C” and another susceptible haplotype “A_T”. However, no significant difference was found between the two susceptible haplotypes. As only one line has the haplotype “A_C” in the winter wheat panel, the pairwise comparison of this haplotype was excluded.

**Fig. 4:**
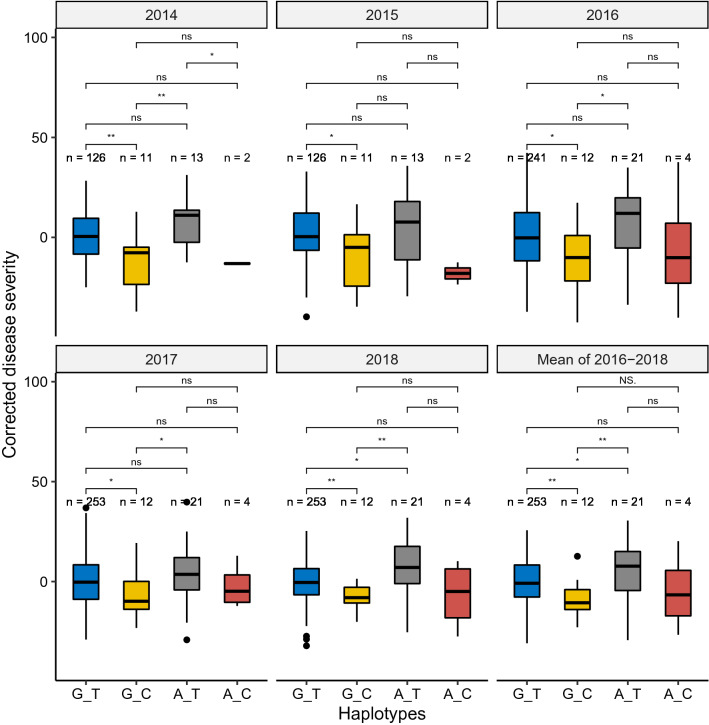
Haplotype analysis of the QTL *QSnb.nmbu-2AS* in the spring wheat panel over five years field trial and mean of three years from 2016 to 2018 (Bottom right) determined by the Wilcox test. ns: *p* > 0.05; *: *p* <  = 0.05; **: *p* <  = 0.01; ***: *p* <  = 0.001

The same haplotype analysis was also carried out for the spring wheat panel (Fig. 4, Table S6). Interestingly, significant differences between resistant haplotype “G_C” and susceptible haplotype “G_T” were consistent across all tested years including the across-year mean from 2016 to 2018. Significant differences between haplotype “G_C” and the susceptible haplotype “A_T” were also observed in all tested years except the year 2015. In addition, there were significant differences (*p* < 0.05) between the susceptible haplotypes “G_T” and “A_T” in year 2018 and the mean of 2016 to 2018.

### Stacking resistant alleles

**Fig. 5:**
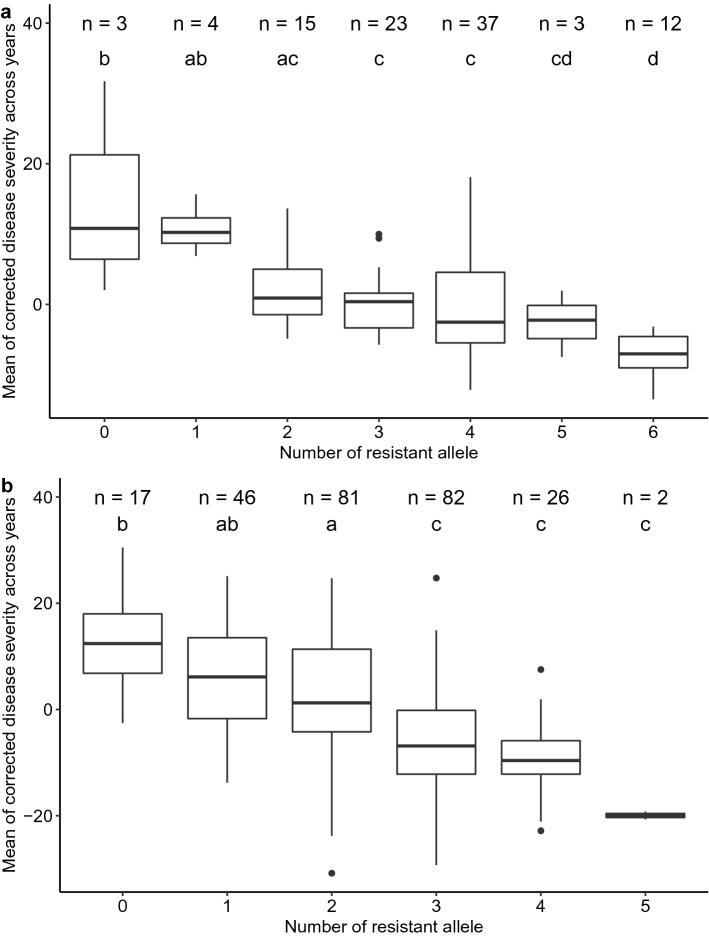
Boxplots showing effects of stacking resistant alleles. **a** Stacking resistant alleles of six selected QTL associated with SNB in the winter wheat panel. **b** Stacking resistant alleles of five selected QTL associated with SNB in the spring wheat panel. Same letter on boxplots indicates no significant difference in mean of disease severities between groups by Tukey`s HSD test (*p* < 0.05)

Markers used for stacking resistant alleles are listed in Table S3. Figure 5a shows the result of stacking resistant alleles in the winter wheat panel. There were three lines, Rida, Redcoat, and Xi19, which had no resistant allele. Among these, the UK cultivar Xi19 was the most susceptible line to SNB in this association mapping panel. The remaining lines were grouped to have between 1 and 6 resistant alleles. A decreasing trend of disease severity could be observed when the number of resistant alleles was increasing. From Fig. 5a, at least two more resistant alleles were required to obtain significant differences in the average disease severities between groups. Similar results could also be seen for the spring wheat panel (Fig. 5b). The more resistant alleles the lines carried, the lower their disease severities (Fig. 5b). However, no significant differences were found between groups with 3, 4, and 5 resistant alleles in the spring wheat panel.

## Discussion

### Important QTL for adult-plant resistance/susceptibility

A review by Ruud and Lillemo (2018) showed that only a few of the known NE-sensitivity loci had been proven to show effects in adult-plant susceptibility in the field. In our study, we found that some NE-sensitivity loci were among the most important susceptibility QTL in adult plants, which were significantly identified in at least one environment (Table 3). Ruud et al. (2018) showed that sensitivity to ToxA was common in the Norwegian spring wheat panel and it was associated with high disease severity in the field. However, markers linked to the susceptibility locus *Tsn1* were not significantly detected above the threshold in the GWAS study using the subset of the NMBU spring wheat panel (Ruud et al. 2019). In this study, a larger panel of spring wheat lines was assessed for SNB field resistance from 2016 to 2018, and significant markers were co-located with the ToxA sensitivity locus *Tsn1* (Friesen et al. 2009), as the *Tsn1*-linked SSR marker *fcp001* (546 Mbp) (Friesen et al. 2006) and another significant SNP marker, *AX-89422431* (547 Mbp), were both located close to the *Tsn1* locus (546.8 Mbp) on the physical map (Faris et al. 2010)*.* Another well-documented SNB sensitivity locus, *Snn2*, which showed an effect on adult-plant susceptibility (Friesen et al. 2009), was also identified as robust QTL *QSnb.nmbu-2DS* during one year in the spring wheat panel and in one year and the mean across years in the winter wheat panel (Table 3). The QTL *QSnb.nmbu-1BS* was likely caused by the Tox1 susceptibility locus *Snn1*. Among the three significant markers detected for this QTL, the *Snn1*-linked SSR marker *fcp618* (0.7 Mbp) (Reddy et al. 2008) was detected as a putative QTL by the across-year mean, while marker *AX-95154820* (4 Mbp) was above the − log_10_(*p*) 4.0 threshold in 2019 and was detected as a putative QTL in 2016 and the across-year mean. The last marker, *BS00067247_51* (4 Mbp), which was detected as a putative QTL by the across year mean, has also been reported as a *Snn1*-linked marker by Cockram et al. (2015). The *Snn3*-*B1* linked marker *BS00091519_51* (Friesen et al. 2007; Ruud et al. 2019) was detected as QTL *QSnb.nmbu-5BS.1* in year 2016 and the across-year mean also using our winter wheat panel (Table 3). In addition, the common spring and winter wheat QTL on the short arm of chromosome 1A (*QSnb.nmbu-1AS.1*) could be the Tox4 sensitivity locus *Snn4* (Abeysekara et al. 2012, 2009). Flanking markers of *Snn4* were located to 3.84–4.20 Mbp of the physical map, while our 1AS QTL was located to 1 Mbp (Table 3). Ruud et al. (2019) also found a QTL on 1AS by seedling inoculation with isolate 201618 however, culture filtrate infiltration of the same isolate could not induce a sensitivity reaction on the *Snn4* differential line (AF89). The significant marker *RAC875_c30657_82* of that 1AS QTL from Ruud et al. (2019) was located at 7.18 Mbp on the physical map which was away from the *QSnb.nmbu-1AS.1* compared to the *Snn4* locus (Table 3). However, it was close to our second common QTL, *QSnb.nmbu-1AS.2,* between winter and spring wheat panels on chromosome 1A (9–14 Mbp).

Besides the NE sensitivity loci, we also compared other identified QTL in this study with previously published QTL. We found that our robust QTL *QSnb.nmbu-2AS* (4–24 Mbp) might overlap with two adult-plant leaf blotch QTL *QSnl05.daw-2A* and *QSnl04.daw-2A* (Francki et al. 2018)*,* which were located at 2–20 and 15–16 Mbp on the reference genome, respectively. In addition, seedling resistance QTL *QSnb.niab-2A.1* (0.8–2.4 Mbp) reported by Lin et al. (2020) and *Qsnb.cur-2AS2* (2.3–3.8 Mbp) reported by Phan et al. (2016) were also mapped close to this region. *QSnb.nmbu-2AL,* located at 742–743 Mbp, might overlap with the seedling resistance QTL by Hu et al. (2019), as the flanking markers of that seedling resistance QTL were located at 735 and 752 Mbp, respectively. Jighly et al. (2016) detected a glume blotch resistance QTL, of which one significant marker was located at 14 Mbp and close to *QSnb.nmbu-3BS* (17–22 Mbp) in our study. *QSnb.nmbu-5BL.2* was located at 514 Mbp, which might overlap with the QTL *QInf.nmbu-5B.1* (509–516 Mbp) detected by Lin et al. (2021) using culture filtrate infiltration of isolate 203649 which did not contain any known effectors. Moreover, many QTL such as *QSnb.nmbu-2BS* and *QSnb.nmbu-3AL* were detected for the first time in this study without any previously published SNB-related QTL located in the nearby region (Table 3).

### Comparing QTL detected from the spring and winter wheat panels

The SNB QTL that we identified varied from year to year and only a few QTL were detected in more than two environments (years). We were able to identify ten QTL which were significant in both the spring and winter wheat panels. However, except for *QSnb.nmbu-2AS* and *QSnb.nmbu-3AL*, none of the other QTL showed significance above the stringent threshold of − log10(*p*) 4.0 in both the winter and spring wheat panels. This indicated that, for field resistance, SNB QTL only showed minor effects and were significantly influenced by the environments. Interactions between *P. nodorum* and wheat depend on both NEs expressed in the pathogen and the hosts` genetic backgrounds (Peters Haugrud et al. 2019). In our study, naturally infected straw was used as inoculum and the pathogen population might vary from year to year due to *P. nodorum*`s mixed reproduction system. Therefore, the significant QTL might also vary according to the local pathogen population with varying frequencies and expression levels of NEs.

### Haplotype analysis

As mentioned in the results section, in winter wheat *QSnb.nmbu-2AS* was only significantly detected in year 2017 and the across-year mean. However, strong haplotype effects of our 2AS QTL were detected also in years when the QTL was below the threshold such as 2018 and 2019. In addition, the historical data from previous years provided evidence of the strong haplotype effects (Fig. 3). Only 15.7% of the lines in the winter wheat panel carry this resistant haplotype, and most of the lines with the resistant haplotype “G_C” are of German origin. This group includes the winter wheat cultivars Jenga and Kuban which are used as resistant checks for SNB field trials in Norway. Most of the Norwegian and Swedish cultivars and breeding lines belong to the larger group with the susceptible haplotype “G_T”.

Haplotype effects of this QTL were also significant in the spring wheat panel, although the resistant haplotype is rare (4.0%) in this panel (Fig. 4). Most spring wheat lines with the resistant haplotype “G_C” originate from CIMMYT and have very good SNB resistance. One of the lines in this group was “Milan” and two additional lines in this resistant group had “Milan” in their pedigree, suggesting “Milan” to be one of the resistance sources. The majority of lines in our spring wheat panel carry the susceptible haplotype. Interestingly, a few recent breeding lines from the Norwegian breeding company Graminor carry the resistance haplotype, including the variety “Seniorita”, which was released in 2014. This might indicate that the beneficial QTL allele is a recent introduction in Norwegian spring wheat breeding.

### Stacking resistant alleles

For both the spring and winter wheat panels, significant effects of reducing SNB field severity were seen by combining resistant alleles of the most consistent QTL. As shown in Fig. 5, the combination of five to six resistant alleles resulted in an overall reduction of SNB field severity by about 15 percentage units in both panels. This indicates that marker-assisted selection can be applied in breeding even though only a few QTL with minor effects are available for SNB resistance. By combining enough resistant loci, it is possible to reduce the SNB disease severity significantly.

## Conclusion

Our results illustrated the challenge of selecting reliable QTL for improving SNB resistance in wheat breeding. Only a few QTL were detected across years. However, the haplotype analysis confirmed the robustness of the QTL on chromosome 2AS. As the resistant haplotype was rare in both Norwegian winter wheat and spring wheat lines, integrating this resistance allele in the local wheat germplasm would help to improve the SNB resistance. In addition, by stacking resistance alleles, the SNB disease severity was significantly reduced, indicating that marker-assisted allele pyramiding can be a promising strategy for reducing SNB susceptibility. However, QTL validations in the field using different plant materials and testing in different environments are needed to reduce the unnecessary cost of integrating inconsistent QTL.

## Supplementary Information

Below is the link to the electronic supplementary material.

Supplementary file1 (DOCX 27 KB)

Supplementary file2 (PDF 1883 KB)

Supplementary file3 (PDF 2501 KB)

Supplementary file4 (PDF 6 KB)

Supplementary file5 (PDF 7 KB)

Supplementary file6 (PDF 7 KB)

Supplementary file7 (TXT 11362 KB)

Supplementary file8 (TXT 4119 KB)

Supplementary file9 (CSV 16 KB)

Supplementary file10 (CSV 7 KB)

## Data Availability

The genotypic and phenotypic data generated during this study are included in this published article [and its supplementary information files].
