# Supplementary material for: Genome-wide association mapping of septoria nodorum blotch resistance in Nordic winter and spring wheat collections
Source: Theor Appl Genet. 2022 Sep 23;135(12):4169–82. doi: 10.1007/s00122-022-04210-z (PMC9734210; doi:10.1007/s00122-022-04210-z)
Supplement: Supplementary file 1 — Supplementary file1 (DOCX 27 KB) [file 122_2022_4210_MOESM1_ESM.docx]

Fig. S1 QQ plots of marker-trait associations for corrected SNB disease severity in the spring wheat panel. a) year 2016, b) year 2017, c) year 2018, d) mean of three years. Markers analyzed by the FarmCPU model were indicated in red dots, while markers analyzed by the MLM+PCs model were indicated in blue dots.

Fig. S2 QQ plots of marker-trait associations for corrected SNB disease severity in the winter wheat panel. a) year 2016, b) year 2017, c) year 2018, d) year 2019 e) mean of four years. Markers analyzed by the FarmCPU model were indicated in red dots, while markers analyzed by the MLM+PCs model were indicated in blue dots.

Fig. S3 Pairwise comparison for means of corrected disease severities in the spring panel between two alleles of the six significant MTAs selected for allele stacking. The Wilcox test was used to determine the differences between two groups. ns: p > 0.05; *: p <= 0.05; **: p <= 0.01; ***: p <= 0.001; ****: p <= 0.0001.

Fig. S4 Pairwise comparisons for means of corrected disease severities in the winter wheat panel between two alleles of the six significant MTAs selected for allele stacking. The Wilcox test was used to determine the differences between two groups. ns: p > 0.05; *: p <= 0.05; **: p <= 0.01; ***: p <= 0.001; ****: p <= 0.0001.

Fig. S5 Histograms of distributions of corrected SNB disease severities for (a) the spring wheat panel from years 2016 to 2018 and the mean over three years. (b) the winter wheat panel from years 2016 to 2019 and the mean over four years.

Table S1 Significant markers associated with SNB field resistance of the spring wheat panel during years 2016 to 2018 and the mean over three years. Physical positions were based on blastn results against the reference genome IWGSC RefSeq v1.0 (International Wheat Genome Sequencing et al. 2018)

| Trait | Chromosome | SNP | Physical position (Mbp) | effect | -log10(p) |
| --- | --- | --- | --- | --- | --- |
| 2016 | 1A | RAC875_c42700_264 | 1 | 3.98 | 3.02 |
|  | 1A | wsnp_Ex_c7252_12453079 | 14 | -6.32 | 3.71 |
|  | 1A | wsnp_Ex_c19717_28713417 | 14 | -6.67 | 3.73 |
|  | 1A | RAC875_c23587_271 | 14 | 6.47 | 3.73 |
|  | 2A | AX-94646265 | 743 | 5.12 | 2.79 |
|  | 2B | wsnp_Ex_c326_636368 | 1 | -3.93 | 2.77 |
|  | 2B | RAC875_rep_c109471_154 | 4 | 3.98 | 2.75 |
|  | 2B | RAC875_rep_c115433_378 | 4 | 3.93 | 2.77 |
|  | 2B | AX-158575300 | 4 | 4.09 | 2.95 |
|  | 2D | AX-95146929 | 2 | 4.63 | 2.89 |
|  | 2D | AX-94903439 | 2 | 4.94 | 3.20 |
|  | 3A | AX-94463375 | 588 | -4.82 | 2.82 |
|  | 3B | AX-109283105 | 18 | 3.69 | 2.83 |
|  | 3B | AX-111657425 | 660 | 4.99 | 3.03 |
|  | 3B | AX-111455746 | 664 | -4.11 | 2.73 |
|  | 6A | IAAV151 | 574 | -4.82 | 2.90 |
|  | 7A | Tdurum_contig62357_527 | 611 | -4.23 | 3.66 |
|  | 7A | AX-94387533 | 618 | -3.82 | 2.93 |
|  | 7A | BobWhite_c30461_131 | 648 | -3.83 | 2.97 |
| 2017 | 1A | Excalibur_c12215_352 | 46 | -1.66 | 2.99 |
|  | 1B | tplb0029b01_1022 | 329 | -3.46 | 3.82 |
|  | 1B | BobWhite_c2027_215 | 641 | 1.89 | 3.09 |
|  | 1D | BS00085821_51 | 449 | -2.08 | 3.23 |
|  | 2B | wsnp_Ra_c1660_3275687 | 1 | -1.94 | 2.87 |
|  | 3A | wsnp_Ra_c29624_38979654 | 735 | 2.90 | 2.91 |
|  | 3B | AX-108940705 | 545 | 1.70 | 2.85 |
|  | 3B | wsnp_JD_c5643_6802088 | 618 | -2.53 | 5.15 |
|  | 4A | wsnp_Ex_c13953_21831752 | 737 | -1.68 | 2.95 |
|  | 4B | Excalibur_c37565_709 | 612 | -2.96 | 3.52 |
|  | 5A | wsnp_Ex_c10231_16783750 | 49 | 4.21 | 5.77 |
|  | 5B | AX-89422431 | 547 | 3.30 | 7.92 |
|  | 5B | AX-94559013 | 662 | -1.98 | 2.93 |
|  | 5B | Tdurum_contig35470_227 | 664 | 1.89 | 2.81 |
|  | 5B | RAC875_c29488_56 | 664 | -1.90 | 2.88 |
|  | 6A | Excalibur_c1492_1282 | 436 | 3.24 | 4.73 |
|  | 7A | AX-158559574 | 611 | 4.13 | 7.51 |
|  | 7B | AX-94730987 | 106 | 1.79 | 2.74 |
|  | 7B | wsnp_BE443010B_Ta_2_1 | 519 | -3.84 | 4.64 |
| 2018 | 1A | RAC875_c23587_271 | 14 | 2.41 | 3.88 |
|  | 1B | AX-95245523 | 16 | -1.23 | 2.91 |
|  | 1D | AX-94981729 | 412 | -1.79 | 3.91 |
|  | 2A | Excalibur_c41459_96 | 12 | -3.19 | 4.75 |
|  | 3A | AX-158523630 | 724 | -2.38 | 6.71 |
|  | 3B | AX-158579373 | 22 | 1.61 | 3.53 |
|  | 3B | BS00077967_51 | 750 | -2.26 | 3.88 |
|  | 5A | AX-95106872 | 10 | -3.52 | 3.35 |
|  | 5A | AX-89444521 | 488 | -3.85 | 5.25 |
|  | 5A | Ex_c4208_839 | 573 | -1.56 | 3.53 |
|  | 5B | AX-95632177 | 600 | 2.58 | 4.01 |
|  | 5D | Excalibur_c91745_337 | 542 | -1.83 | 4.12 |
|  | 6A | Kukri_c494_1413 | 615 | -1.89 | 2.90 |
|  | 6A | Kukri_c494_1479 | 615 | -1.91 | 2.91 |
|  | 6B | Ku_c106365_329 | 475 | -2.10 | 5.62 |
|  | 6D | AX-95143298 | 468 | -1.65 | 3.30 |
|  | 7A | AX-158553506 | 33 | 1.88 | 4.73 |
|  | 7A | AX-158567020 | 522 | -1.86 | 3.16 |
|  | 7B | AX-94682021 | 534 | -5.98 | 7.90 |
| Mean | 1A | wsnp_Ex_c19717_28713417 | 14 | -2.69 | 3.54 |
|  | 1B | AX-94856891 | 675 | -2.16 | 3.76 |
|  | 2A | RAC875_c20979_234 | 742 | -2.60 | 5.25 |
|  | 2B | AX-158575300 | 4 | 1.90 | 4.00 |
|  | 2D | AX-158522481 | 77 | -1.55 | 3.61 |
|  | 3A | AX-94468289 | 509 | 2.13 | 3.51 |
|  | 3A | AX-94619572 | 511 | -2.00 | 3.15 |
|  | 3A | wsnp_Ex_c15475_23757972 | 689 | -2.20 | 3.62 |
|  | 3B | AX-89731276 | 500 | -2.03 | 3.46 |
|  | 3B | AX-94776694 | 504 | 1.93 | 3.19 |
|  | 3B | AX-111482352 | 506 | 2.00 | 3.15 |
|  | 3B | AX-110517995 | 523 | 2.19 | 3.37 |
|  | 3B | wsnp_JD_c5643_6802088 | 618 | -2.82 | 6.75 |
|  | 3D | wsnp_Ex_c15036_23203474 | 85 | 4.21 | 3.26 |
|  | 5B | Tdurum_contig12540_72 | 370 | -2.15 | 3.23 |
|  | 5B | fcp001 | 546 | -2.55 | 3.60 |
|  | 5B | BS00078784_51 | 668 | 2.60 | 4.73 |
|  | 7A | AX-158559574 | 611 | 3.63 | 8.11 |
|  | 7D | AX-111916790 | 201 | 1.70 | 3.16 |

Table S2 Significant markers associated with SNB field resistance of the winter wheat panel during years 2016 to 2019 and the mean over four years. Physical positions were based on blastn results against the reference genome IWGSC RefSeq v1.0 (International Wheat Genome Sequencing et al. 2018)

| Trait | Chromosome | SNP | Physical position (Mbp) | effect | -log10(p) |
| --- | --- | --- | --- | --- | --- |
| 2016 | 1B | AX-95154820 | 4 | 4.67 | 3.68 |
|  | 4A | wsnp_BF474615A_Ta_1_1 | 582 | -6.76 | 3.67 |
|  | 5A | GENE-3324_338 | 4 | 4.47 | 4.01 |
|  | 5B | BS00091519_51 | 7 | 4.52 | 4.02 |
|  | 5B | RAC875_c31482_513 | 341 | -9.70 | 4.35 |
|  | 5B | BS00023803_51 | 350 | 11.04 | 4.94 |
|  | 5B | Kukri_c23070_350 | 350 | -9.71 | 4.35 |
|  | 5B | TA008675-0589 | 353 | -9.71 | 4.35 |
|  | 5B | BS00002191_51 | 354 | 9.71 | 4.35 |
|  | 5B | Kukri_c36789_230 | 356 | 9.71 | 4.35 |
|  | 5B | wsnp_Ex_c2132_4004831 | 356 | -9.71 | 4.35 |
|  | 5B | wsnp_Ex_c5632_9904112 | 356 | -9.71 | 4.35 |
|  | 5B | wsnp_Ku_c7199_12444840 | 357 | 9.71 | 4.35 |
|  | 5B | wsnp_Ex_c40022_47169698 | 357 | -11.04 | 4.94 |
|  | 5B | IAAV3825 | 358 | 9.71 | 4.35 |
|  | 5B | Excalibur_c60683_908 | 366 | -11.04 | 4.94 |
|  | 5B | wsnp_Ex_c53011_56395185 | 369 | 9.71 | 4.35 |
|  | 5D | AX-95175890 | 310 | 8.45 | 3.81 |
|  | 7B | tplb0025i03_2004 | 352 | -8.75 | 3.67 |
| 2017 | 2A | Excalibur_c15379_1305 | 3 | 4.96 | 4.09 |
|  | 2A | RFL_Contig4030_493 | 4 | -4.76 | 3.91 |
|  | 2A | Excalibur_c62893_629 | 4 | 4.99 | 3.96 |
|  | 2A | Kukri_c31776_1621 | 8 | 4.99 | 3.96 |
|  | 2A | Excalibur_c10442_1911 | 11 | 5.03 | 4.04 |
|  | 2A | GENE-1273_59 | 11 | 5.03 | 4.04 |
|  | 2A | IAAV8501 | 12 | -4.99 | 3.96 |
|  | 2A | Kukri_c23195_266 | 12 | 5.03 | 4.03 |
|  | 2A | BS00022760_51 | 15 | 5.01 | 4.01 |
|  | 2A | AX-94510523 | 15 | -4.88 | 3.83 |
|  | 2A | Kukri_c12648_434 | 19 | -4.99 | 3.96 |
|  | 2A | AX-158573223 | 24 | -5.62 | 3.84 |
|  | 2A | TA003766-0683 | 24 | -4.99 | 3.96 |
|  | 2A | BS00010696_51 | 689 | -8.24 | 4.12 |
|  | 2B | AX-94893801 | 19 | -4.99 | 3.96 |
|  | 2B | BobWhite_c12426_84 | 22 | 4.99 | 3.96 |
|  | 2B | GENE-1123_531 | 35 | 4.92 | 3.88 |
|  | 2D | Excalibur_c25599_358 | 8 | 4.99 | 3.96 |
|  | 2D | Excalibur_c18324_390 | 8 | 5.03 | 4.04 |
| 2018 | 1A | AX-110004070 | 9 | 5.66 | 2.74 |
|  | 1A | RAC875_rep_c104335_293 | 9 | 5.66 | 2.74 |
|  | 1A | RAC875_rep_c72805_651 | 9 | -5.66 | 2.74 |
|  | 1A | RAC875_c19509_174 | 212 | 4.60 | 2.87 |
|  | 1B | BS00067247_51 | 298 | -5.98 | 4.74 |
|  | 3A | AX-110989461 | 712 | 6.11 | 3.60 |
|  | 3A | AX-158523648 | 722 | -3.38 | 3.06 |
|  | 3A | AX-158533085 | 722 | -3.33 | 3.11 |
|  | 3B | AX-158563148 | 17 | -4.20 | 2.81 |
|  | 3B | AX-158541608 | 562 | -3.04 | 2.97 |
|  | 4A | RAC875_c91464_170 | 512 | 2.87 | 2.81 |
|  | 4B | Kukri_c49506_396 | 16 | -3.39 | 3.30 |
|  | 4B | AX-158583166 | 17 | -3.00 | 2.71 |
|  | 5D | BobWhite_c14323_288 | 409 | 4.22 | 2.84 |
|  | 6B | Tdurum_contig28247_226 | 718 | -4.67 | 3.04 |
|  | 6B | TA002907-0816 | 720 | 4.89 | 2.94 |
|  | 6B | BS00011795_51 | 721 | 4.55 | 2.82 |
|  | 6B | Excalibur_c55484_393 | 721 | 6.38 | 4.83 |
|  | 7A | AX-158590739 | 37 | 4.13 | 2.92 |
| 2019 | 1A | GENE-0014_822 | 1 | -1.74 | 2.59 |
|  | 1A | AX-95094547 | 13 | 2.46 | 3.35 |
|  | 1B | AX-95154820 | 4 | 3.23 | 4.39 |
|  | 1D | D_GDS7LZN02IXNP1_255 | 2 | -1.74 | 2.59 |
|  | 1D | Kukri_c26168_713 | 2 | -1.74 | 2.59 |
|  | 1D | RAC875_c10925_1887 | 2 | -1.74 | 2.59 |
|  | 1D | RAC875_c20883_693 | 2 | -1.74 | 2.59 |
|  | 1D | Kukri_c36351_64 | 2 | 1.90 | 3.02 |
|  | 2D | TA020686-0548 | 62 | -2.15 | 3.04 |
|  | 3A | TG0146 | 722 | 2.40 | 5.30 |
|  | 3B | wsnp_Ex_c8208_13870372 | 742 | -2.65 | 5.58 |
|  | 4A | AX-108808218 | 4 | -1.76 | 3.07 |
|  | 4A | AX-94387070 | 5 | 1.72 | 2.98 |
|  | 4A | AX-158558358 | 715 | 2.59 | 3.43 |
|  | 5A | AX-158542802 | 458 | 3.54 | 5.91 |
|  | 5A | AX-110506047 | 589 | 2.28 | 2.60 |
|  | 5B | BS00023803_51 | 350 | 3.47 | 3.16 |
|  | 5B | IAAV5683 | 514 | 2.95 | 3.72 |
|  | 6A | AX-94544731 | 11 | 1.50 | 2.72 |
|  | 6B | TA002907-0816 | 720 | 3.09 | 4.80 |
|  | 7A | AX-94484859 | 85 | 1.89 | 2.75 |
|  | 7D | AX-158568416 | 23 | 1.33 | 3.76 |
|  | Unmapped | AX-158567462 | - | -1.75 | 2.97 |
| Mean | 1B | fcp618 | 1 | -3.09 | 3.73 |
|  | 1B | BS00022504_51 | 4 | 3.11 | 3.62 |
|  | 1B | AX-95154820 | 4 | 3.28 | 3.97 |
|  | 2A | Excalibur_c10442_1911 | 11 | 3.37 | 3.06 |
|  | 2A | GENE-1273_59 | 11 | 3.37 | 3.06 |
|  | 2A | Kukri_c23195_266 | 12 | 3.37 | 3.06 |
|  | 2A | AX-94510523 | 15 | -3.37 | 3.08 |
|  | 2A | AX-158573241 | 16 | 3.39 | 3.14 |
|  | 2A | BS00027484_51 | 17 | -3.37 | 3.10 |
|  | 2A | AX-158573223 | 24 | -4.02 | 3.29 |
|  | 2B | AX-94739692 | 35 | -2.78 | 3.27 |
|  | 2D | Excalibur_c18324_390 | 8 | 3.37 | 3.06 |
|  | 3A | BobWhite_rep_c49102_169 | 535 | 3.52 | 3.04 |
|  | 3A | Kukri_c34195_357 | 537 | -3.52 | 3.04 |
|  | 3A | Kukri_c80104_809 | 539 | 3.52 | 3.04 |
|  | 5B | BS00091519_51 | 7 | 2.69 | 3.15 |
|  | 5B | BS00023803_51 | 350 | 5.81 | 3.04 |
|  | 5B | wsnp_Ex_c40022_47169698 | 357 | -5.81 | 3.04 |
|  | 5B | Excalibur_c60683_908 | 366 | -5.81 | 3.04 |
|  | 5B | IAAV5683 | 514 | 4.99 | 4.03 |

Table S3 Markers used for stacking resistant alleles

| Marker | Chromosome | Physical map position (Mbp) | Association panel |
| --- | --- | --- | --- |
| RAC875_c20979_234 | 2A | 742 | Spring wheat |
| AX-158575300 | 2B | 4 | Spring wheat |
| wsnp_JD_c5643_6802088 | 3B | 618 | Spring wheat |
| AX-89422431 | 5B | 547 | Spring wheat |
| BS00078784_51 | 5B | 668 | Spring wheat |
| AX-158559574 | 7A | 611 | Spring wheat |
| AX-95154820 | 1B | 4 | Winter wheat |
| Excalibur_c15379_1305 | 2A | 3.5 | Winter wheat |
| Excalibur_c18324_390 | 2D | 8 | Winter wheat |
| BS00091519_51 | 5B | 6.6 | Winter wheat |
| BS00023803_51 | 5B | 350 | Winter wheat |
| IAAV5683 | 5B | 514 | Winter wheat |

Table S4 The -log10(p) values of the 0.1 percentile threshold in each environment

| Trait | Threshold (-log10(p)) |
| --- | --- |
| Spring wheat 2016 | 2.72 |
| Spring wheat 2017 | 2.73 |
| Spring wheat 2018 | 2.89 |
| Spring wheat mean | 3.11 |
| Winter wheat 2016 | 3.6 |
| Winter wheat 2017 | 3.82 |
| Winter wheat 2018 | 2.71 |
| Winter wheat 2019 | 2.63 |
| Winter wheat mean | 3.04 |

Table S5: Mean of correct disease severities for each haplotype of *QSnb.nmbu-2AS* in winter wheat panel in each environment

| Haplotype | 2010 | 2011 | 2012 | 2014 | 2015 | 2016 | 2017 | 2018 | 2019 | Mean of 2016-2019 |
| --- | --- | --- | --- | --- | --- | --- | --- | --- | --- | --- |
| G_T | 0.18 | -0.24 | -0.45 | -0.06 | 0.12 | 1.06 | 1.19 | -0.38 | 1.18 | 0.82 |
| G_C | -21.96 | -9.67 | -19.56 | -7.61 | -18.83 | -4.76 | -8.08 | -3.53 | -4.09 | -5.12 |
| A_T | -5.01 | -7.12 | -12.10 | 4.82 | -4.95 | 0.91 | -1.11 | 3.53 | -0.94 | 0.74 |
| A_C | NA | NA | NA | NA | NA | -5.02 | -15.98 | -7.25 | -2.70 | -7.74 |

Table S6: Mean of correct disease severities for each haplotype of *QSnb.nmbu-2AS* in spring wheat panel in each environment

| Haplotype | 2014 | 2015 | 2016 | 2017 | 2018 | Mean of 2016-2018 |
| --- | --- | --- | --- | --- | --- | --- |
| G_T | 0.55 | 1.30 | -0.20 | 0.14 | -0.14 | -0.12 |
| G_C | -12.45 | -10.14 | -10.51 | -7.20 | -7.35 | -8.35 |
| A_T | 6.24 | 3.93 | 6.15 | 4.16 | 6.82 | 5.71 |
| A_C | -13.08 | -18.01 | -5.70 | -2.22 | -6.78 | -4.90 |
