# Supplementary figures and images for: Genome-wide association mapping of septoria nodorum blotch resistance in Nordic winter and spring wheat collections
Source: Theor Appl Genet. 2022 Sep 23;135(12):4169–82. doi: 10.1007/s00122-022-04210-z (PMC9734210; doi:10.1007/s00122-022-04210-z)

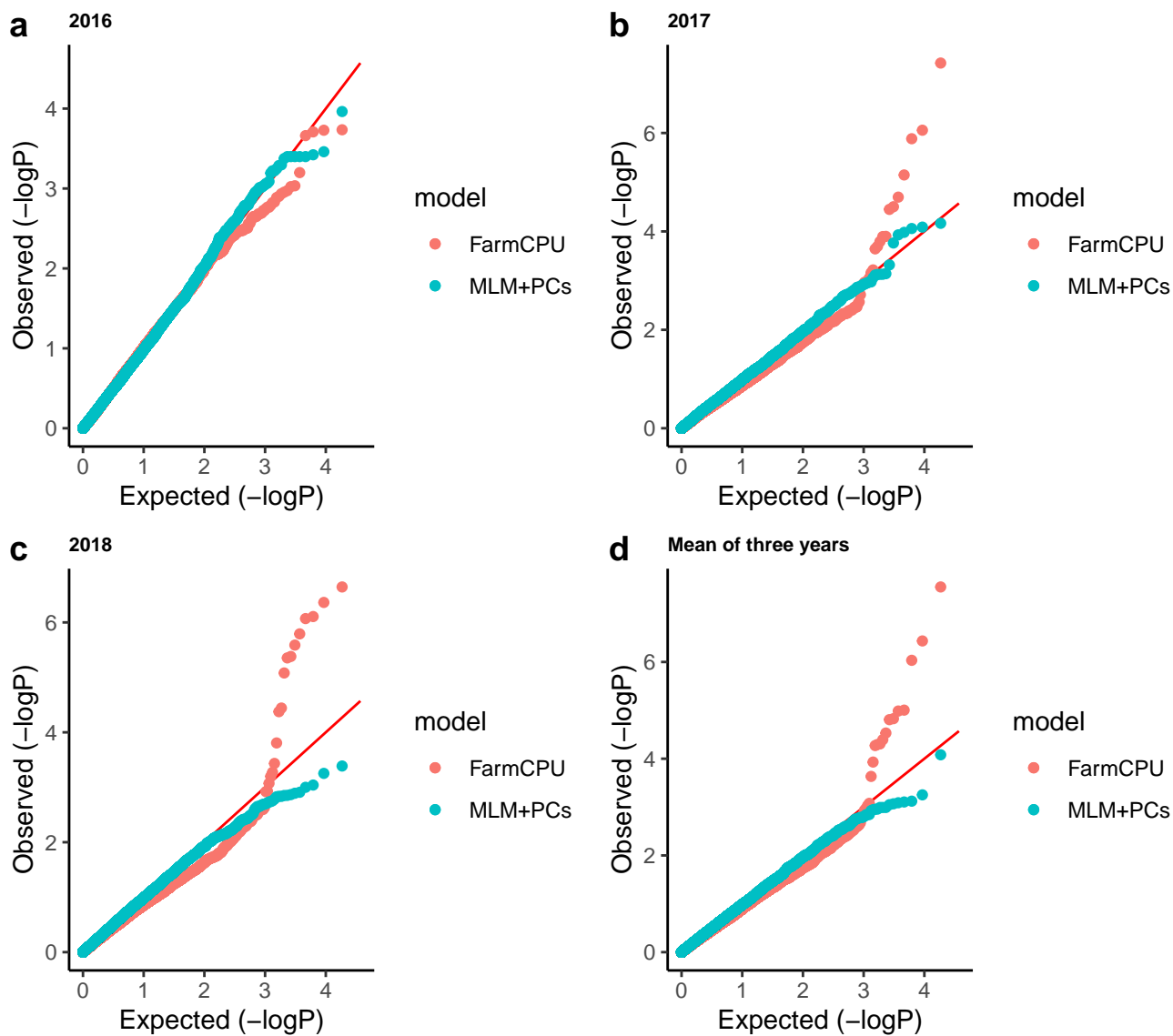

Supplement: Supplementary file 2 — Supplementary file2 (PDF 1883 KB) [file 122_2022_4210_MOESM2_ESM.pdf]

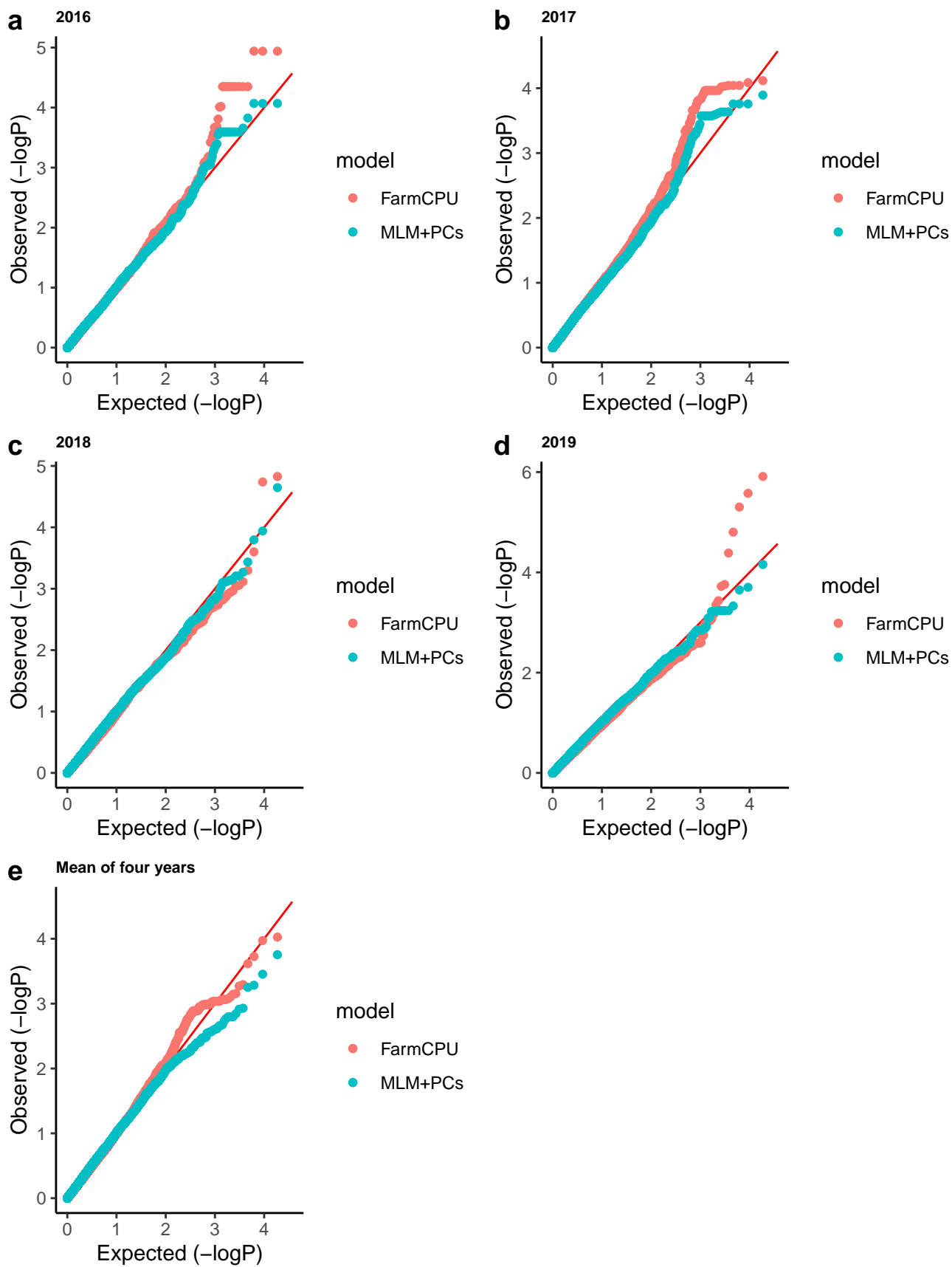

Supplement: Supplementary file 3 — Supplementary file3 (PDF 2501 KB) [file 122_2022_4210_MOESM3_ESM.pdf]

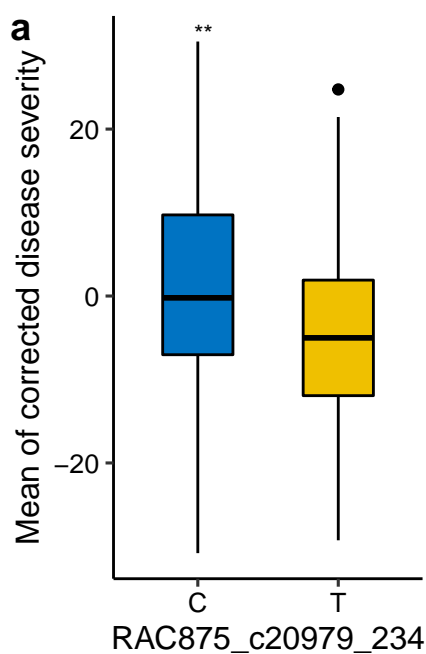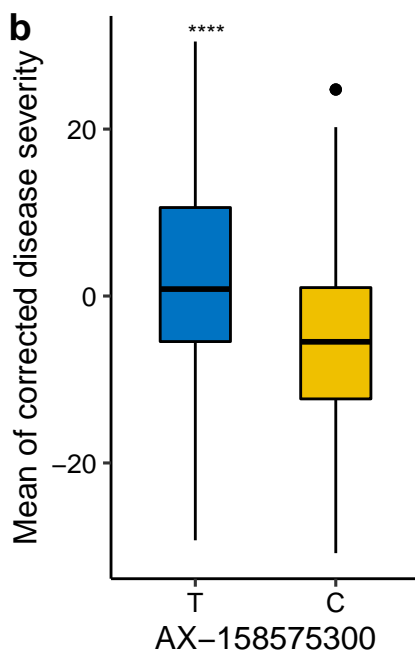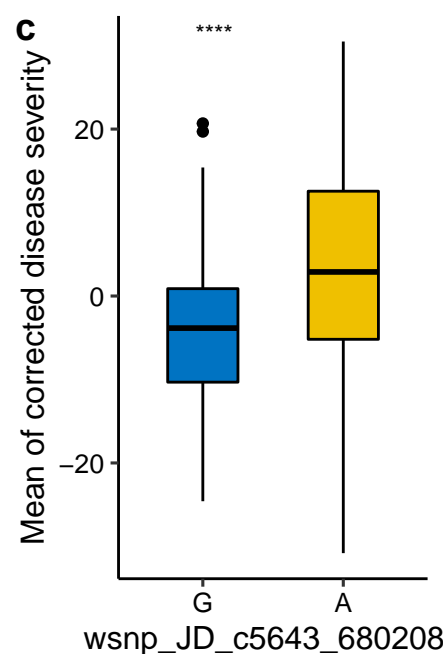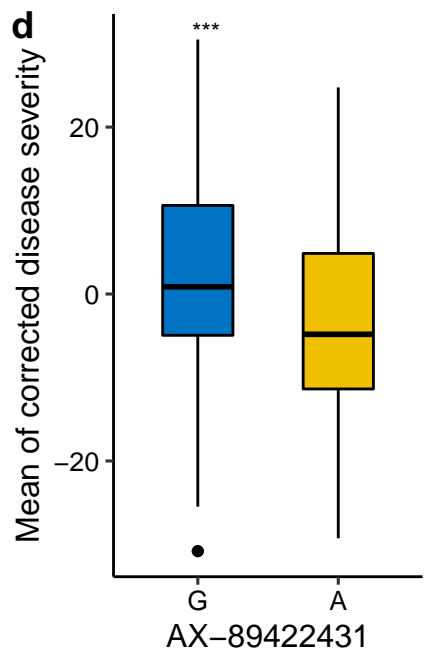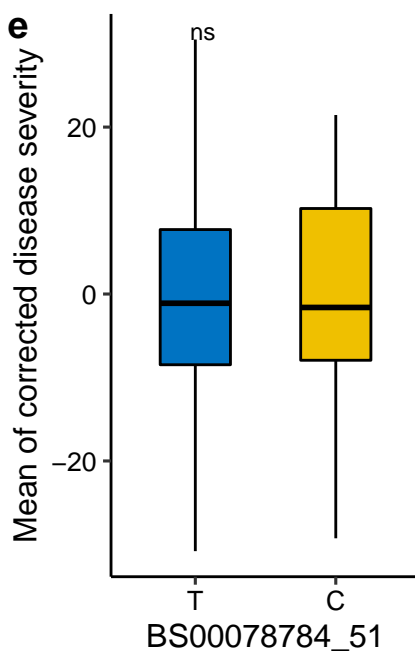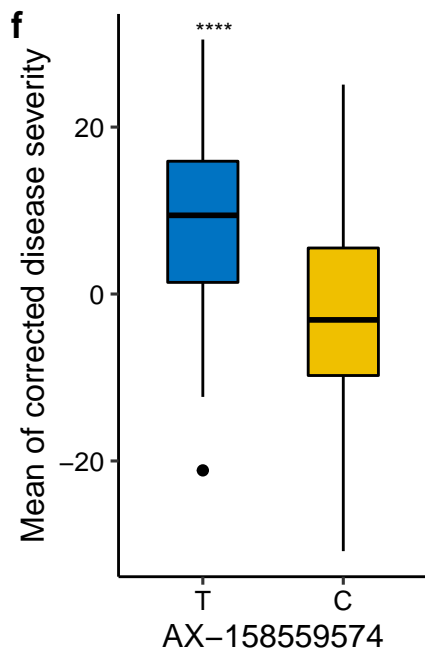

Supplement: Supplementary file 4 — Supplementary file4 (PDF 6 KB) [file 122_2022_4210_MOESM4_ESM.pdf]

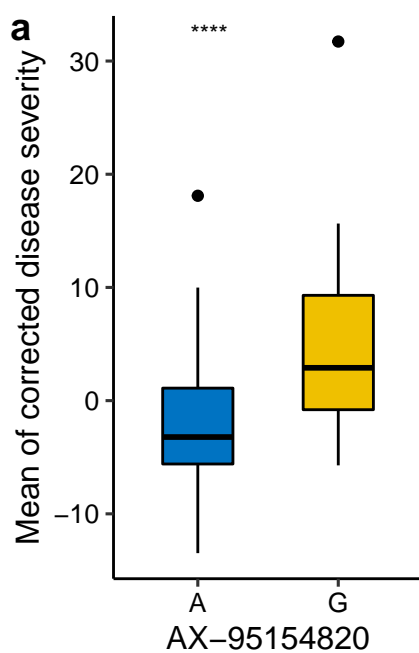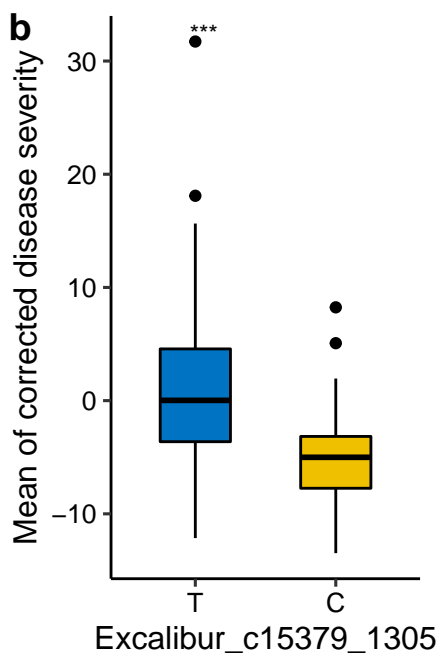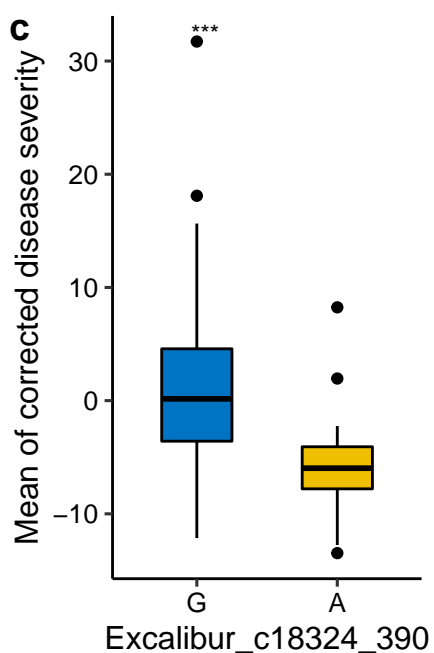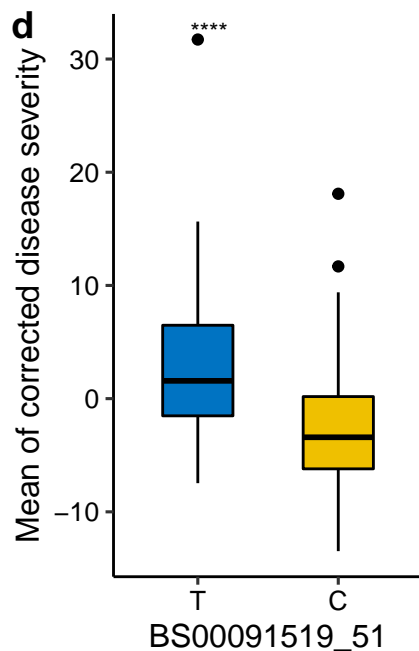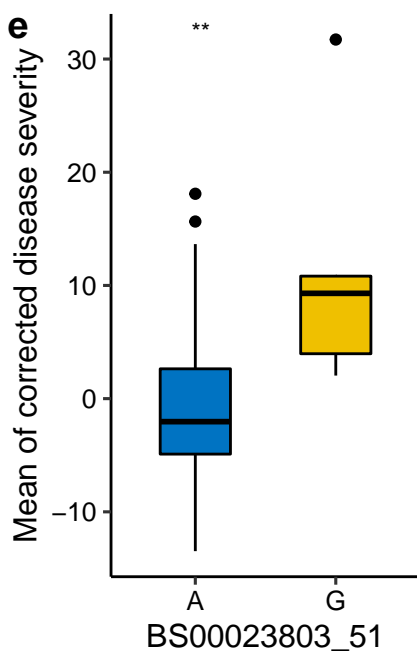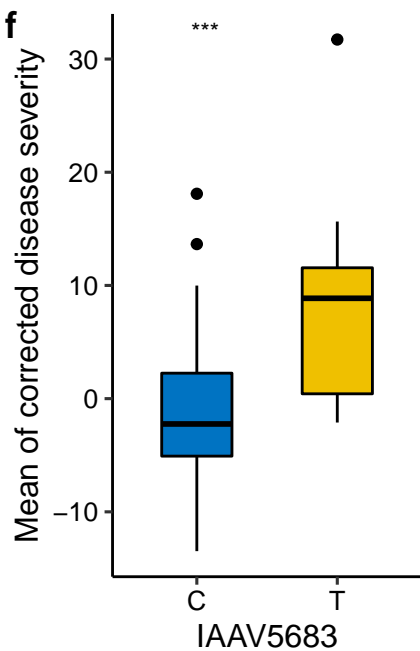

Supplement: Supplementary file 5 — Supplementary file5 (PDF 7 KB) [file 122_2022_4210_MOESM5_ESM.pdf]

a)

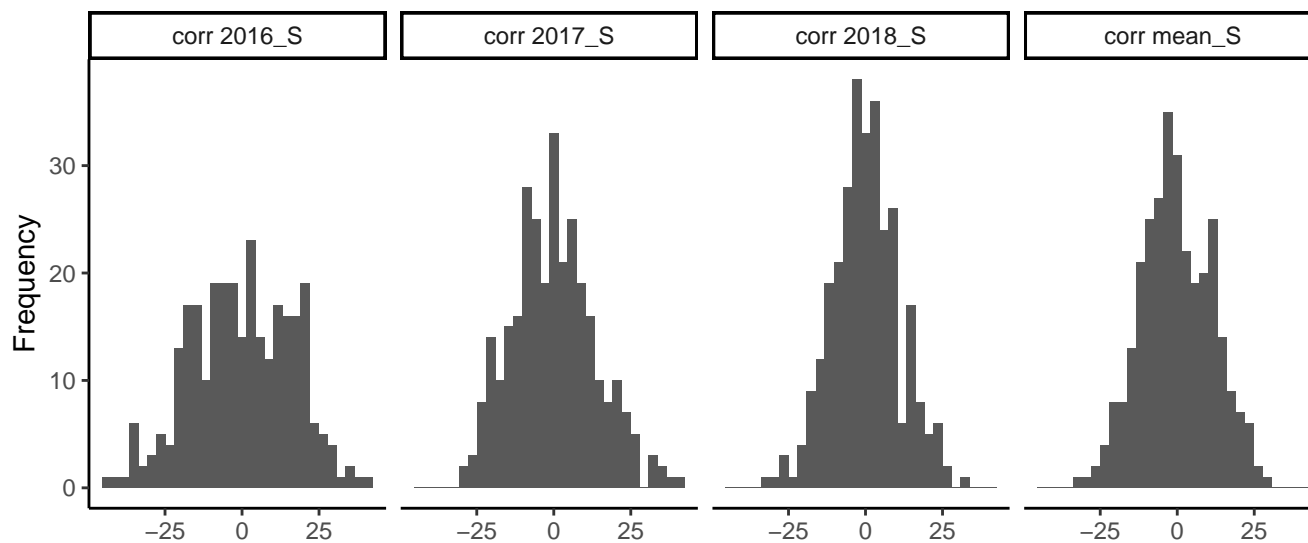

b)

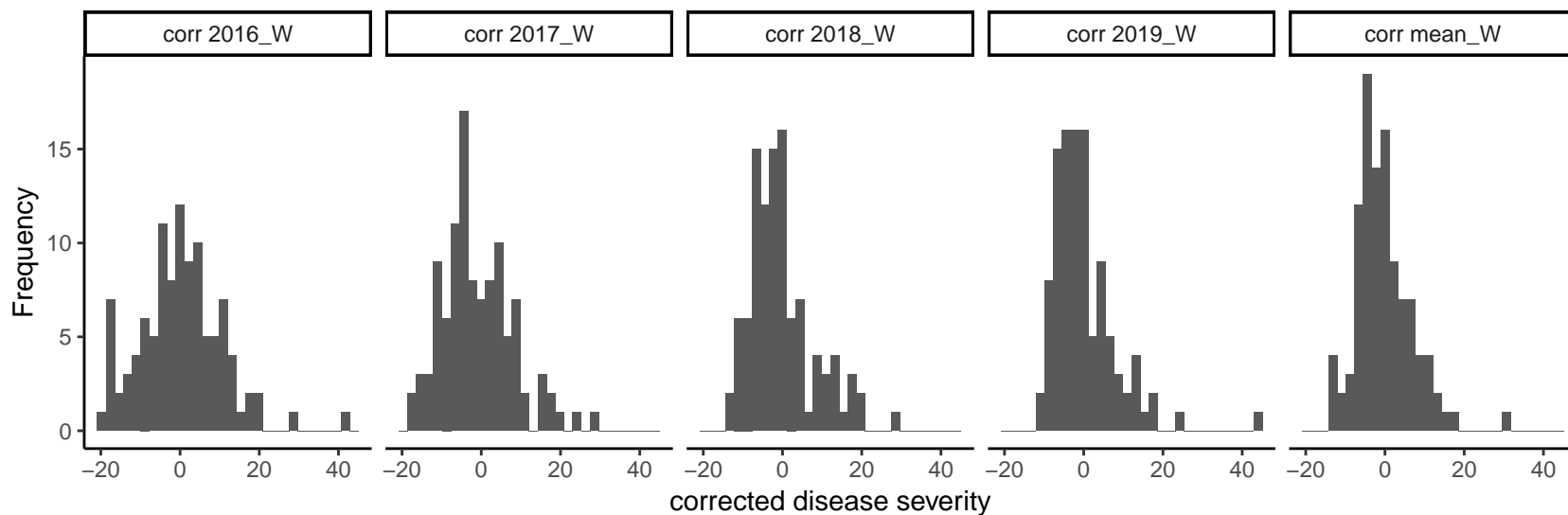

Supplement: Supplementary file 6 — Supplementary file6 (PDF 7 KB) [file 122_2022_4210_MOESM6_ESM.pdf]
